# Patterns in the longitudinal oropharyngeal microbiome evolution related to ventilator-associated pneumonia

**DOI:** 10.1186/s13756-019-0530-6

**Published:** 2019-05-22

**Authors:** Rami Sommerstein, Tobias M. Merz, Sabine Berger, Julia G. Kraemer, Jonas Marschall, Markus Hilty

**Affiliations:** 1 Department of Infectious Diseases, Bern University Hospital, University of Bern, Freiburgstrasse; 4, 3010 Bern, Switzerland; 2 Department of Intensive Care, Bern University Hospital, University of Bern, Bern, Switzerland; 3 Cardiovascular Intensive Care Unit, Auckland City Hospital, Auckland, New Zealand; 4 Institute for Infectious Diseases, University of Bern, Friedbühlstrasse 51, 3001 Bern, Switzerland

**Keywords:** Ventilator-associated pneumonia, Intensive care, Oropharyngeal and tracheal microbiome, Infection prevention, Nosocomial pneumonia

## Abstract

**Background:**

The aim of the study was to evaluate the composition and the temporal evolution of the oropharyngeal microbiome in antibiotic-naïve patients requiring mechanical ventilation and to gain new insights into the pathogenesis of ventilator-associated pneumonia (VAP).

**Methods:**

Prospective, observational single-center nested case-control study. Patients with acute critical illness and anticipated duration of mechanical ventilation > 4 days were eligible. We took oropharyngeal swabs (and if available, tracheal secretions) daily, starting at the day of intubation. The microbiota was characterized by 16S rRNA high-throughput sequencing and compared between patients developing VAP versus controls.

**Results:**

Five patients developed VAP. In three patient the causative pathogens were Enterobacteriaceae and in two *Haemophilus influenzae*. Locally weighted polynomial regression suggested that the within diversity (=alpha) was lower in Enterobacteriaceae VAP patients between days two to five of mechanical ventilation when compared to controls. Detection of Enterobacteriaceae in the oropharynx occurred on day two of follow-up and consisted of a single operational taxonomic unit in 2/3 patients with enterobacterial VAP.

**Conclusions:**

In acutely-ill patients who developed enterobacterial VAP the causative pathogen gained access to the oropharynx early after starting mechanical ventilation and outgrew the commensal members of the microbiome. Whether a specific pattern of the oropharyngeal microbiome between days three to five of mechanical ventilation may predict VAP enterobacterial VAP has to be evaluated in further studies.

**Electronic supplementary material:**

The online version of this article (10.1186/s13756-019-0530-6) contains supplementary material, which is available to authorized users.

## Background

Ventilator-associated pneumonia (VAP) is the most common hospital-acquired infection in intensive care units (ICU), and is associated with prolonged mechanical ventilation, increased mortality, higher costs, and increased antibiotic consumption [1–4]. Microaspiration of the oropharyngeal microbiota represents an important pathway leading to VAP [4, 5]. Most measures to decrease VAP rates aim at preventing the transfer of pathogens from the oropharynx to the lungs [6]. A meta-analysis showed that oral decontamination was the only intervention to reduce mortality in the context of VAP [7]. Reliable markers to predict the onset of VAP do not exist. Continuous surveillance cultures may predict the microbial cause of a VAP for *Streptococcus pneumoniae*, *Acinetobacter* spp., and/or other multi-drug resistant Gram-negative bacteria [8, 9] and therefore help with the selection of appropriate antibiotic therapy. However, surveillance cultures do not predict the time of onset of VAP, nor provide essential information for preventive measures [9].

The human microbiome is defined as the ecological community of commensal, symbiotic and pathogenic microorganisms that inhibit body spaces [10]. The human lung microbiome and its role in health and disease has gained greater attention among researchers in the last 10 years [11–13]. The lower respiratory tract has historically been considered sterile, but recent evidence supports the concept that a distinct microbiota of the lower respiratory tract is present both in health and in various respiratory diseases [14–16]. Some authors argue that the pharyngeal microbiome may have a protective role in respiratory tract infections and that artificial reinforcement of microbiome homeostasis would be an option to prevent invasion of species causing infections [17]. In contrast, during influenza infection, the respiratory microbiome seems to undergo only very discrete changes [18].

The use of longitudinal study designs is essential to gain an understanding of the variation of the microbiome within individual subjects and during development of VAP [19]. A common approach for assessing community changes is the evaluation of the within-subject changes in bacterial diversity (=alpha; the variety and abundance of organisms in the microbiome) over time. However, it is also essential to use an in-between diversity measure (=beta; the dissimilarity between multiple microbiomes/samples) that captures changes in microbiome composition rather than just diversity [19].

More recently, the sputum microbiome was found to be indicative of clinical outcomes in lung diseases such as cystic fibrosis and COPD [20–22].

Previous longitudinal analyses of respiratory tract microbiota have provided some insight into the pathogenesis of VAP in critically ill patients [23, 24]. 16S rRNA gene sequencing of endotracheal aspirate samples allowed a broader look of bacterial communities [24]. Dysbiosis of microbial communities in the respiratory tract was most pronounced in patients who already had developed VAP [23]. However, no longitudinal study has yet been performed in acutely ill patients using daily oropharyngeal swabs with paired tracheal secretions. Our goal was to describe the longitudinal dynamics of the oropharyngeal and tracheal microbiota and stratify this evolution for the onset or absence of a VAP. Our main study hypotheses were i) VAP is associated with disturbed oropharyngeal microbiota, ii) distinct operational taxonomic units of the Enterobacteriaceae family have a specific dynamic pattern of gaining access to the oropharynx during the course of mechanical ventilation and iii) the causative Enterobacteriaceae may outgrow the commensal members of the microbiome.

## Methods

### Study design and setting

The study was designed as a prospective, single-center nested case-control study and was performed at the Department of Intensive Care Medicine (ICU) at Bern University Hospital, Switzerland. The ICU is a 60-bed unit admitting > 6500 patients per year and is the sole provider of intensive care for adults at the hospital, handling medical, surgical and trauma patients. More than 3500 patients per year require mechanical ventilation. Subjects were screened for inclusion between December 2015 and November 2016. The study was conducted in compliance with the study protocol, the current version of the *Declaration of Helsinki*, the *International Council for Harmonisation-Good Clinical Practice* as well as all national legal and regulatory requirements.

### Participants

Consecutive patients admitted to the ICU due to cranio-cerebral trauma, stroke or subarachnoid hemorrhage, and patients with cardiogenic shock were assessed for study inclusion, as prior antibiotic treatment tends to be rare in these patients. Patients were screened by the study team within 24 h after oral intubation for eligibility. Inclusion criteria were: age between 18 and 80 years, and anticipated duration of mechanical ventilation longer than 48 h as determined by the intensivist in charge (with the aim of identifying as many patients as possible who will eventually be ventilated for at least 4 days). Predefined exclusion criteria were chronic immunosuppressive therapy or comorbidities associated with immunosuppression (neutropenia < 0.5 G/l; active leukemia or lymphoma; HIV with CD4 < 200 cells/μl; splenectomy patients; < 4 weeks post-transplant; cytotoxic chemotherapy; high-dose steroids > 2 weeks with prednisone equivalent > 20 mg/d or > 1 week with > 40 mg/die); systemic antibiotic therapy in the last 3 months (except perioperative prophylaxis); active infection upon study screening that required immediate or deferred (within 48 h) systemic antibiotic therapy.

Eligible patients were unable to give consent for the study at the time of enrollment because of mental incapacity due to the underlying medical condition and mechanical ventilation. Before a patient was enrolled, a physician who was not participating in the study confirmed that the interests of the patient were safeguarded and that all inclusion criteria and no exclusion criteria were present. Inclusion occurred after requesting study consent from a patient representative. Patient’s informed consent was sought in a later phase, as soon as the patient’s condition had improved sufficiently. We aimed at including 30 patients to accrue 5–8 VAP cases, as the predicted rate was between 20 and 30%. Overall, ten subjects were included for further processing of the samples (i.e., microbiome evaluation). This included the VAP cases and control patients ventilated for more than four days.

### Clinical variables

The intensivists prospectively collected variables for study patients. These included: Age, gender, SAPS II score upon ICU admission, date of ICU admission, previous and new antibiotic treatment (start and stop date, class, cumulative dose), duration of mechanical ventilation, tracheotomy, duration of ICU stay, degree of respiratory failure at beginning and end of mechanical intubation (PaO2/FiO2), and predominant parameter settings of the mechanical ventilation. All parameters were transferred into a spreadsheet by the ICU’s study nurse team at the end of each study day.

### Outcomes

*Clinical* VAP was defined as a patient with a new or progressive pulmonary infiltrate on chest radiography (according to the radiology report). Also, two of the following three criteria were required: body temperature higher than 38 °C, leukocyte count greater than 12 G/L or lower than 4 G/L, and/or purulent respiratory secretions within 48-h before/after the radiography. A *confirmed* VAP required additional detection of a causative pathogen – either by microbiological culture or via microbiome analysis from a tracheal sample. VAP cases were then subgrouped in enterobacterial vs. *Haemophilus influenzae*. For the control group, patients with ‘no VAP’ were chosen. We addressed the potential bias of misclassifying cases into VAP and controls by having the diagnostic criteria validated by an independent study nurse. This study nurse was blinded in terms of medical records and medications.

### Microbiome

Sampling: For uniformity reasons, all samples were collected by a study nurse. Oropharyngeal samples were taken using eswabs™ (Copan; Murrieta, CA, USA) and immediately stored at − 80 °C. Initial sampling was performed immediately after inclusion; daily follow-up samples were taken each morning before oral hygiene (teeth brushing followed by oral decontamination with chlorhexidine 0.2%). A final sample was taken together at the time of VAP diagnosis or before extubation. If available, tracheobronchial samples were simultaneously collected in a 50 ml tube and stored at − 80 °C. Additionally, the VAP diagnosis sample was sent for routine microbiological assessment.

Processing: Sample processing was performed as recently described [25]. A detailed description is provided in the Additional file 1. The sequence reads were submitted to the European Nucleotide Archive (accession number: PRJEB26875).

### Statistics

Cases characteristics were summarized in a table. Baseline characteristics were compared with chi-square and Mann-Whitney-U tests, as appropriate.

The sample’s microbiome within-subject diversity (=alpha) was determined using the richness and Shannon diversity indices (*vegan* package; *diversity* function in R [26]). Summary alpha diversities were plotted by applying a LOESS smoother on the increasing sample days [26]. Due to the overall small sample size, the alpha diversity of cases with Enterobacteriaceae VAP was visually compared to *H. influenzae* VAPs and to all subjects without VAP and no statistical test was applied.

The between sample dissimilarity (=beta diversity) was calculated using the *vegdist* function in R [26]. Differences were evaluated by applying T-tests. *P*-values < 0.05 were considered significant. All graphs were generated in R [26].

## Results

### Study patients

**Fig. 1:**
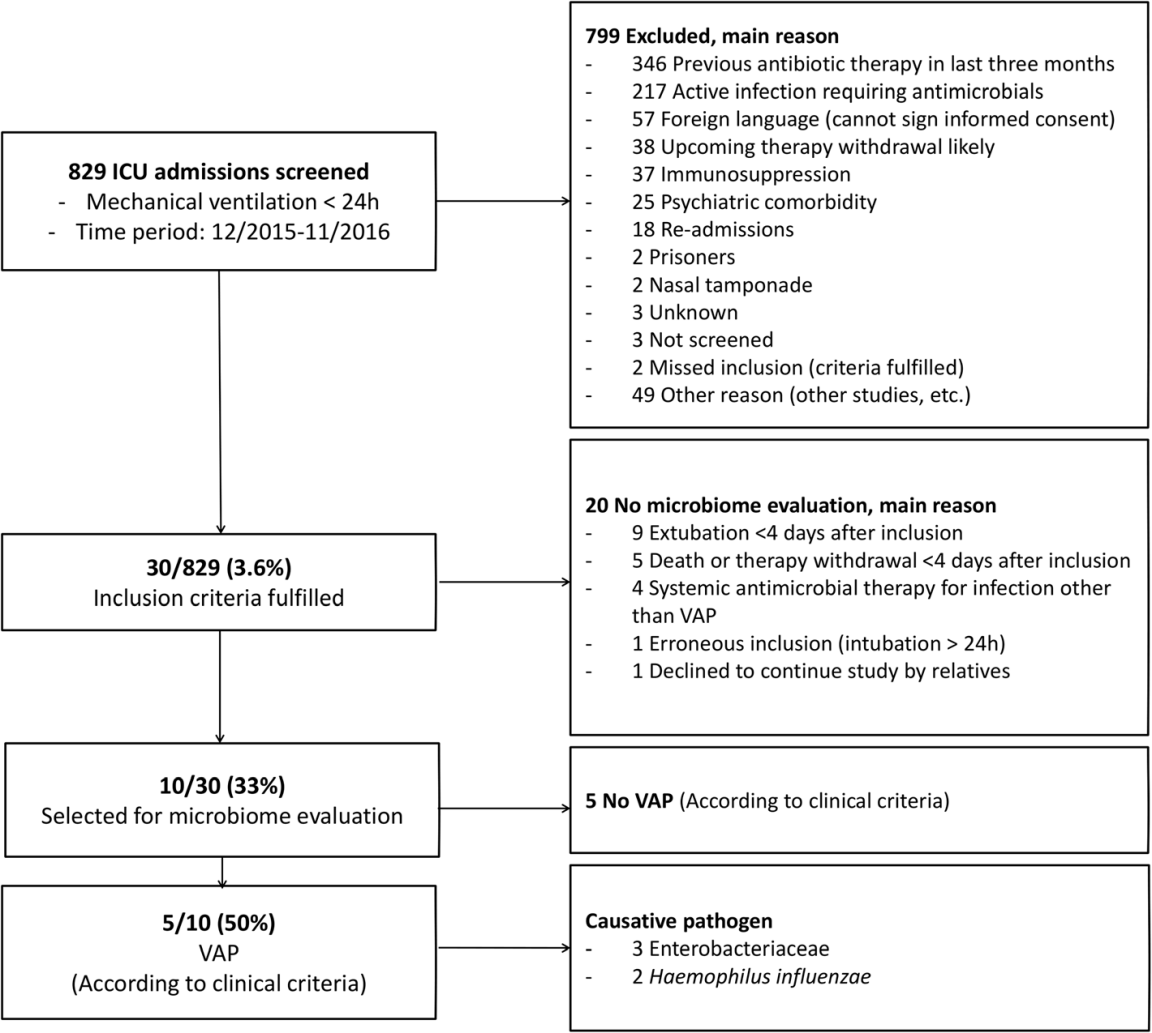
Flowchart. The process of subject screening, inclusion, and selection for microbiome evaluation is shown in the flow chart. Subjects were selected to fulfill possible VAP criteria according to clinical criteria (new infiltrate plus two of the following three: temperature greater than 38 °C, leukocyte count greater than 12 G/L or lower than 4 G/L, or purulent respiratory secretions)

**Fig. 2:**
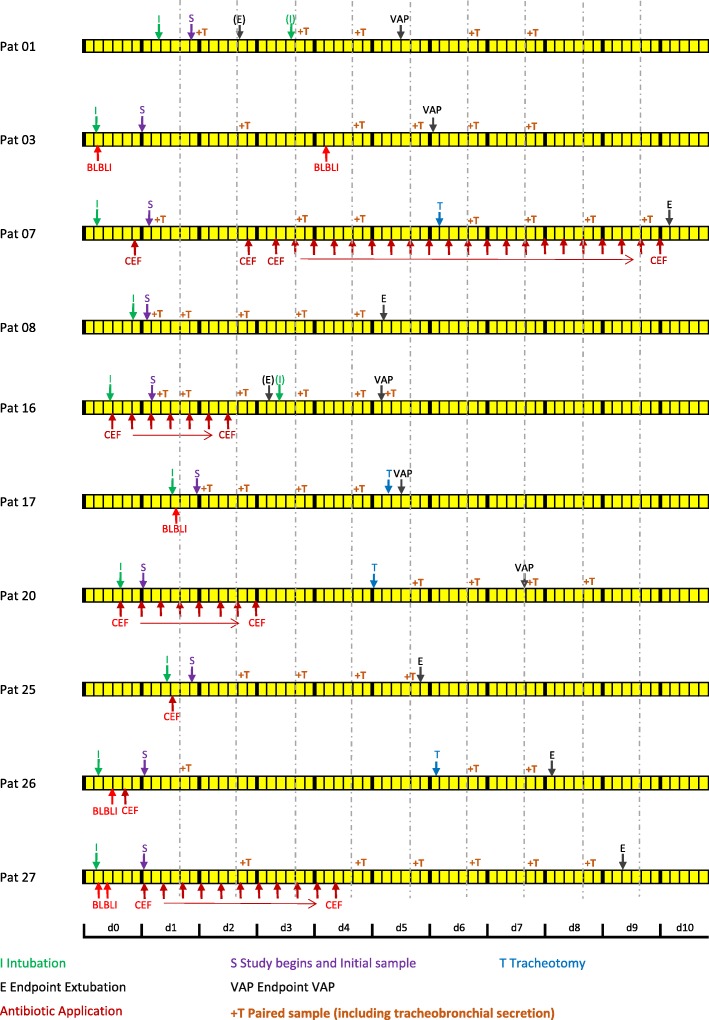
Timeline. For each study patient who underwent microbiome evaluation, a timeline indicates the moment of intubation, study start, endpoint (VAP or extubation), and if performed tracheostomy. Intermittent extubations and reintubations < 24 h were not exclusion criteria and are highlighted in the timeline in parentheses. Each study day lasted from noon to noon of the following day. Follow up sampling was taken at 8 am each day, indicated by dotted, vertical lines, usually starting on day 1. If a paired tracheobronchial secretion was taken in addition to the oropharyngeal sample, times were indicated (+T). In order to align follow-up samples of all study subjects: If the study begin on day 1 was after 8 am, the first follow-up sample was only taken on day 2 (i.e., for subjects 01, 17, 25)

During the study period, 829 ICU admissions were screened for inclusion (Fig. 1). A total of 30 (3.6%) subjects fulfilled inclusion criteria and 10/30 (1.2% of all) nested patients were selected for microbiome evaluation according to the pre-defined criteria. The main reasons for not evaluating the microbiome were early extubation within three days and/or early therapy withdrawal. Of the ten patients, five had a confirmed VAP between days 5–7 after inclusion (Fig. 2). Of these five VAP patients, a microbiological diagnosis was obtained by conventional culture methods in four subjects – and by microbiome analysis in one subject (*Proteus vulgaris* being the causative operational taxonomic unit). The causative pathogens were Enterobacteriaceae in three subjects and *H. influenzae* in two subjects (Table 1).

**Table 1:** Subject characteristics and pneumonia diagnosis

Subject	Sex	Age	Main Diagnosis	SAPS Score 24h after ICU admission	APACHE IV Score 24h after ICU admission	Days enrolled	Sample method	Pathogens detected	Confirmed VAP
1	m	79	Cardiac arrest	82	30	5	TBS	*Haemophilus influenzae*	Y
3	f	56	Subarachnoidal hemorrhage	56	28	7	TBS	*Klebsiella oxytoca*	Y
7	m	69	Polytrauma with intracranial hemorrhage	39	11	10	NA	NA	N
8	m	70	Cardiac arrest	72	26	5	NA	NA	N
16	m	78	Cardiac arrest	77	ND	5	TBS	*Morganella morganii*	Y
17	m	80	Subarachnoidal hemorrhage	79	25	5	TBS	*Haemophilus influenzae*	Y
20	m	71	Intracranial hemorrhage	91	27	7	TBS	*Proteus vulgaris* ^*a*^	Y
25	f	41	Subarachnoidal hemorrhage	47	22	5	NA	NA	N
26	f	45	Subarachnoidal hemorrhage	50	20	8	NA	NA	N
27	m	22	Polytrauma with intracranial hemorrhage	50	25	9	TBS	Not detected	N

^*a*^ *Proteus vulgaris* identified via microbiome analysis

Baseline characteristics of the subjects are listed in Table 1. Baseline characteristics did not significantly differ between VAP cases and controls for gender (*p* = 0.49), diagnosis (*p* = 0.34), and days of enrollment (*p* = 0.42). Patients with VAP were older (median 78, IQR 71–79) than controls (45, 41–69; *p* = 0.03). Also, SAPS scores were higher in VAP (79, 77–82) than controls (50, 47–50; *p* = 0.02), and so were APACHE IV scores (27.5, 26.5–28.5 vs. 22, 20–25; *p* = 0.03).

Figure 2 shows a timeline of each study patient that underwent microbiome evaluation, reporting critical events related to mechanical ventilation, study onset and termination, the sampling strategy and administration of prophylactic antibiotics. Of note, eight of the ten patients received prophylactic antibiotics during intubation because they underwent surgical procedures.

### Sequence analysis, taxonomic assignments and dissimilarity values of oropharyngeal swabs and TBS

In total, 71 oropharyngeal swabs were collected from five VAP patients and five controls, respectively. Additionally, 51 paired and one non-paired TBS were included. For all samples (*n* = 123), we performed subsequent 16S rRNA gene sequencing and received a total of 12,134,989 good quality sequence reads (mean 98,658; 95% CI 83,106-114,211).

**Fig. 3:**
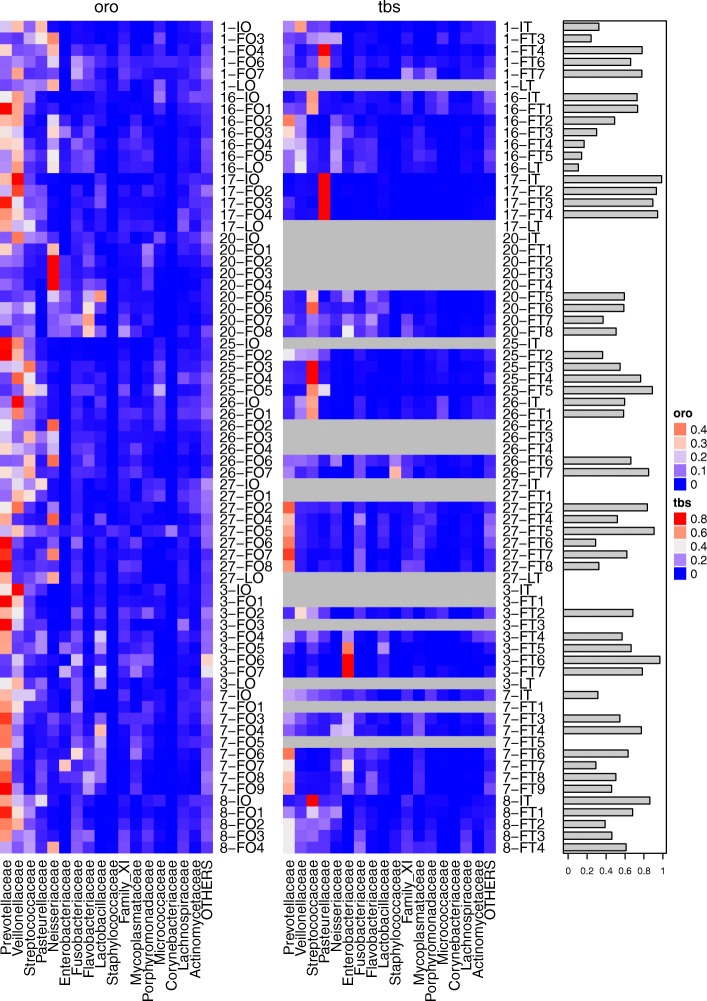
Heatmap of oropharyngeal and tracheobronchial samples. Relative abundances of bacterial communities of the subjects are shown. The left main column are oropharyngeal samples, the middle tracheobronchial and the right shows the relative dissimilarity between the two corresponding samples. Each subcolumn for oropharyngeal (oro) and tracheobronchial (tbs) samples represents family-level taxonomic assignment of the sample’s 16S rRNA gene sequences. The color indicates proportional abundance of the sequences assigned to each bacterial family within the sample. The rows represent individual time points of sampling for the subjects. IO and IT represent the initial oropharyngeal and tracheobronchial samples, respectively. Missing samples are indicated with grey fill

Overall, *dada2* analyses of sequence reads revealed a total of 1688 variants which grouped into 155 bacterial families. As for the latter, the relative abundances of the most abundant families (less abundant families were grouped as ‘others’) are visualized in two heatmaps for each sample (Fig. 3). Prevotellaceae, Veillonellaceae and Streptococcaceae were most frequently identified.

We then created an abundance-based distance matrix and received dissimilarity values for each time point for which two samples were available (right column; Fig. 3). Dissimilarity values of samples from the lower as compared to the upper airways (oropharyngeal swabs) were generally large and there was no clear correlation if VAP patients were compared with controls. This is not true for patient No. 3 for which a decrease in dissimilarity was revealed.

### Access of Enterobacteriaceae to the oropharynx and occurrence of VAP

**Fig. 4:**
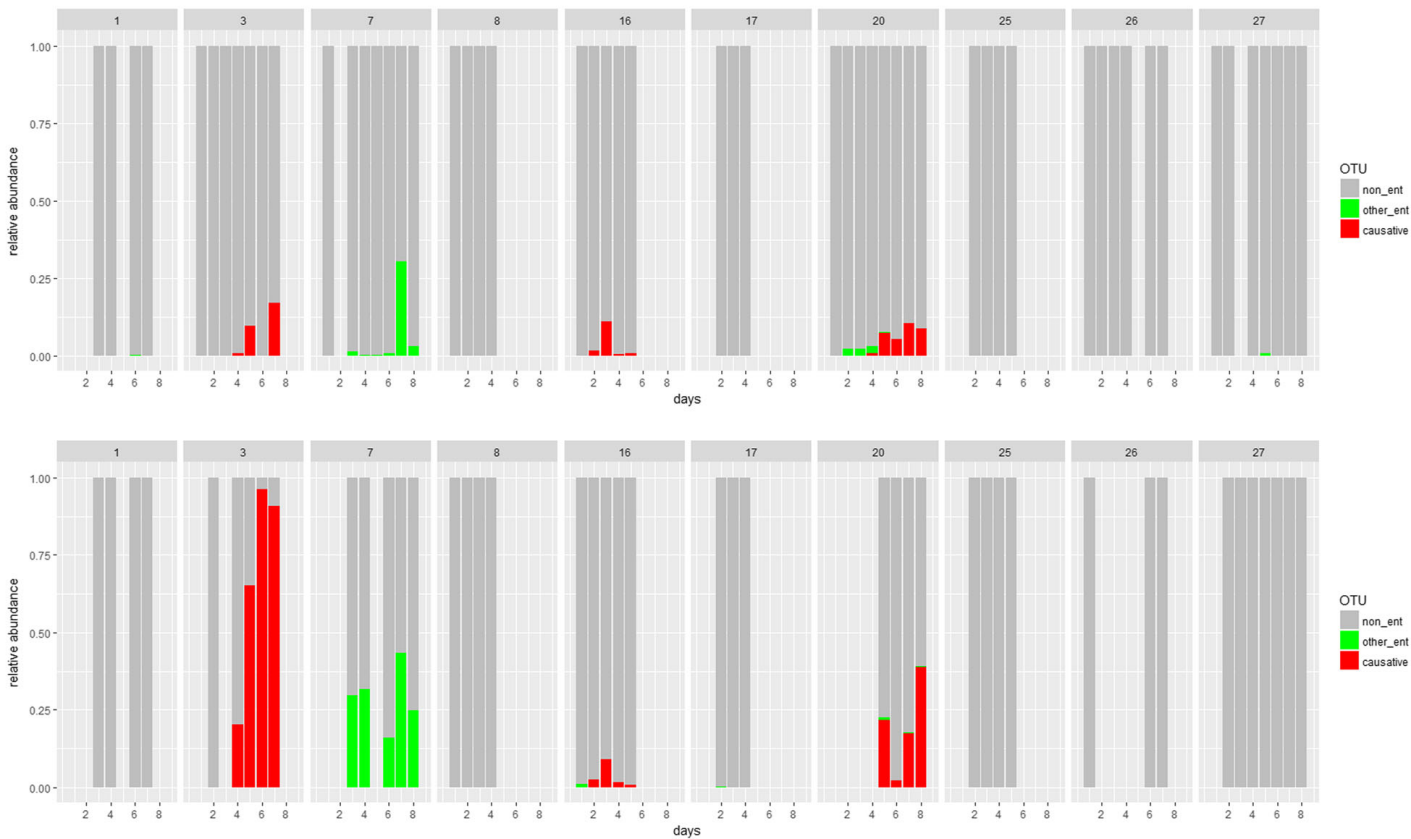
Early access of VAP causative Enterobacteriaceae to the oropharynx. Relative abundance of causative (red) and any other (green) Enterobacteriaceae operational taxonomic units is shown for ten individual study subjects. Results of paired oropharyngeal (upper panels) and tracheobronchial (lower panels) samples are shown. Enterobacteriaceae VAP patients are subjects 3, 16 & 20. The x-axis denotes days post intubation

We detected Enterobacteriaceae in the oropharynx on at least one day during the first five days of mechanical ventilation above 2% (relative abundance) in 40% (4/10) of patients (Fig. 4). Three of these four patients were later diagnosed with an enterobacterial VAP.

In two of these three patients a single enterobacterial operational taxonomic unit was found. The detection of Enterobacteriaceae in the oropharynx correlated with the subsequent colonization of the tracheobronchial system, both in terms of time and operational taxonomic units (Fig. 4).

### Alpha diversity measurements within oropharyngeal samples

**Fig. 5:**
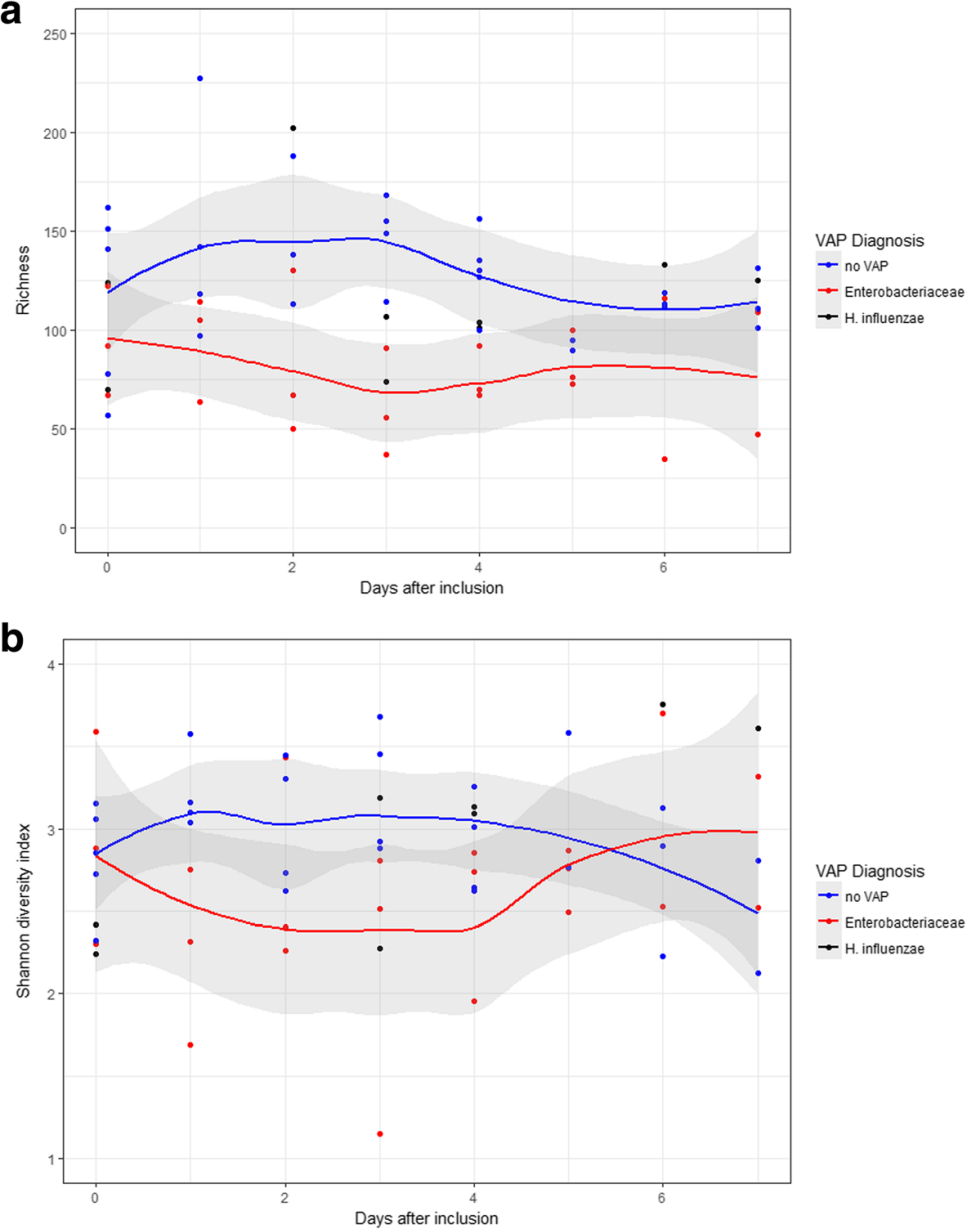
Decrease of oropharyngeal longitudinal alpha diversity in enterobacterial VAP. Longitudinal changes in alpha diversity of the oropharyngeal samples of the individual patients (dots) and by VAP diagnosis. Lines are based on a local polynomial regression fitting (*loess* function in R, grey bands indicate 95% CI). **a** Richness, **b** Shannon diversity index. Note: Summary lines for *H. influenzae* VAP patients were omitted due to low number of cases (*n* = 2)

Next, we examined if longitudinal alpha diversity measurements within the oropharynx differed in patients with enterobacterial VAP from other patients. The "richness" values appeared lower in Enterobacteriaceae VAP patients but not in *H. influenzae* VAP when compared to controls (Fig. 5a). Similarly, the Shannon diversity indices (SDI) for Enterobacteriaceae VAP patients appeared lower than for *H. influenzae* and controls (Fig. 5b).

### Beta diversity measurements in-between oropharyngeal samples

When comparing the oropharyngeal microbiomes in individual patients between the initial sample and day 4, the dissimilarity was not significantly different among patients with Enterobacteriaceae VAP, *H. influenzae* VAP or controls (Additional file 1: Figure S1).

## Discussion

We described the longitudinal dynamics of the oropharyngeal and tracheal microbiota in a highly selected cohort of previously antibiotic-naïve mechanically ventilated patients. Our study identified two important points: firstly, patients with enterobacterial VAP appear to develop a lower within (=alpha) diversity of the oropharyngeal microbiota compared to non-VAP/*H. influenzae* VAP patients. A lowered SDI/richness could therefore be indicative of subsequent VAP occurrence caused by Enterobacteriaceae. Secondly, we showed that VAP causative Enterobacteriaceae accessed the oropharynx early during ventilation (day 2), with a small initial abundance. Of interest, this enterobacterial ‘access’ consisted of a single operational taxonomic unit in 2/3 patients, where the causative bacteria outgrew the residual microbiota and led to VAP. This points to the access of a single clone. These two observations can be used to investigate in a follow-up study with less stringent inclusion criteria whether a defined longitudinal oropharyngeal microbiome pattern between day zero and two of mechanical ventilation may predict VAP.

There are several significant limitations to this study. The most important is the small study size – with only 5 VAP and 5 control patients given the restrictive inclusion criteria.

Of note, the low number of patients led to the post-hoc formation of somehow arbitrary VAP subgroups (enterobacterial versus *H. influenzae*). On the other hand, these criteria allowed us to focus on patients with a relatively undisturbed initial microbiome – as they had been antibiotic-free for the last three months and were not receiving antibiotic treatment for any active infection.

During two years in our academic hospital, we screened every admission to the ICU with conventional prolonged mechanical ventilation for possible inclusion. It must therefore with emphasized that it was not a small study, but a very selective one.

Next, we did not obtain tracheobronchial secretion samples at each time point. Daily induction of sputum was considered unethical in this critically ill cohort and therefore omitted. As a consequence, not all oropharyngeal samples can be compared with their tracheobronchial counterparts and the value of the tracheobronchial longitudinal evaluation was therefore hampered. Next, VAP diagnosis was mainly based on clinical/radiological criteria and did not take the ventilator settings into account. This may have led to a bias in the assignment of cases and controls. Finally, absence of daily chest x-rays could be a reason for further imprecision in the diagnosis of VAP.

Priority was given to the continuous morning sampling before chlorhexidine disinfection for all the follow-up samples every 24 h. This may have influenced the comparability of the dynamic microbiome evolution since repeated morning sampling was not done in relation to the time of intubation. Another limitation was the frequent use of antibiotic prophylaxis *during the study*, which could have had an impact on the composition and the dynamic of the microbiome. Previously it was shown, that long-term macrolide treatment changed the composition of respiratory microbiota and was associated with decreased bacterial richness [27–29]. In a previous VAP study though, it was shown that systemic antibiotic administration were not associated with changes in the alpha diversity [23]. Therefore it is difficult to estimate how much these antibiotics confounded the results.

We interpret our results as important new insight into the pathogenesis of VAP. It was surprising to see that a single causative operational taxonomic unit accesses the oropharynx early on and then outgrows commensal members. This finding may have important implications for VAP diagnosis and prevention: Sampling of the oropharyngeal microbiota around day three to five of mechanical ventilation could be indicative of VAP occurring 2–4 days later. Of note, in 50% of patients with occurrence of a single Enterobacteriaceae no VAP was diagosed over the clinical course. Therefore this occurrence alone is not sufficiently predictive. Co-markers (such as decreased alpha-diversity) could play an additional role. Such markers need to be identified in a more extensive study.

From a preventive perspective, our findings could be interpreted in such a way that efforts to impede Enterobacteriaceae accessing the oropharynx should presumably start immediately after intubation. We think that this finding is generalizable to other settings, as a similar – monoclonal - colonization of Enterobacteriaceae in the airways of ventilated patients has been reported before [30]. Decreased alpha diversity in lower and upper respiratory tract samples in ventilated compared to healthy subjects has also been demonstrated before [24]. Moreover, a decrease in longitudinal alpha diversity in VAP patients versus ventilated controls has been shown before in tracheal aspirates [23, 31, 32]. Therefore, our data expand this finding to the oropharynx, an anatomical region that is probably key in the natural history of VAP development [4, 5]. Our results support the findings of a recent study in which reduced microbial diversity in mechanically ventilated patients was associated with mortality and was suggested to be a biomarker of prognostic value [33].

Our findings should be confirmed in a more extensive study and be compared to data from other critically ill or previously ventilated patients. In the meantime we think that our findings should be considered when planning further studies of predictive diagnostic approaches or preventive strategies.

## Conclusions

In patients who develop enterobacterial VAP the causative pathogen appears to gain access to the oropharynx early after starting ventilation and outgrow the commensal members of the microbiome. Whether a specific pattern of the oropharyngeal microbiome on day three to five of mechanical ventilation may predict VAP enterobacterial VAP has to be evaluated in further studies.

## Additional file

Additional file 1: a) Supplementary Methods (Microbiome); b) Supplementary References; c) Additional file 1: Figures S1 & S2. (DOCX 47 kb)

## Abbreviations

APACHE: Acute Physiology and Chronic Health Evaluation
BLBLI: Betalactam plus betalactam-inhibitor class antibiotic
CEF: 1./2. generation cephalosporin class antibiotic
ICU: Intensive care unit
NA: Not available
ND: Not determined
OTU: Operational taxonomic units
SAPS: Simplified Acute Physiology Score
TBS: Tracheobronchial secretions
VAP: Ventilator-associated pneumonia
