# Supplementary material for: Patterns in the longitudinal oropharyngeal microbiome evolution related to ventilator-associated pneumonia
Source: Antimicrob Resist Infect Control. 2019 May 22;8:81. doi: 10.1186/s13756-019-0530-6 (PMC6530040; doi:10.1186/s13756-019-0530-6)

**Supplementary Material**

**Supplementary Methods**

*Microbiome*

DNA from all samples was extracted using the Qiagen DNA Minikit (Qiagen, Hilden, Germany), following the spin protocol for DNA purification from body fluids. Subsequently, the V4 region of the 16S rRNA gene was amplified using forward (5’-GTGCCAGCMGCCGCGGTAA-3’) and reverse (5’-GGACTACHVGGGTWTCTAAT-3’) primers^1^ and modified with an Illumina adaptor sequence at the 5’ end. PCR cycling conditions were 95 °C for 6 minutes and 35 cycles at 95 °C for 30 seconds, at 59 °C for 30 seconds and then at 72 °C for 1.5 minutes (with a final elongation step at 72 °C for 5 minutes). PCR products were purified by QIAquick PCR purification kit (Qiagen, Hilden, Germany). The samples were semi-quantified using agarose gels and samples with low DNA concentration were additionally quantified using the DNA 7500 kit with an Agilent 2100 Bioanalyzer (Agilent Technologies, Palo Alto, CA). A minimal DNA concentration of 1 ng/µl after PCR and purification was required for subsequent processing, as described before.^2^ As part of our quality control, a clean cotton swab tip was exposed for several seconds during the sampling procedure and processed together with the samples from this study. However, none of the negative control samples were above 1 ng/µl after PCR and purification and they were, therefore, not sent for sequencing. Samples were submitted for indexing and pair-end 2x250 bp sequencing (Reagent Kit v2) on the Illumina MiSeq platform (San Diego, USA).

Analyses: Reads were analysed using the *dada2* package version 1.5.0 and *workflow*^3^ in R version 3.1.2.^4^ We have recently validated *dada2* with the “mothur" pipeline and found a very high agreement.^5^ Forward and reverse reads were trimmed at 200 bp and at 150 bp to remove low quality regions, respectively. The 20 first base pairs and instances of a quality score ≤2 were truncated from all reads. All reads with identical sequences were collapsed. The amplicon errors were corrected using the *dada2* algorithm with default parameters. The denoised output reads were merged and all reads with any mismatches were removed. Sequence variants (SVs) shorter than 245 or longer than 257 base pairs where removed as were chimeras. Taxonomy was assigned using the *assignTaxonomy* function, which implements the RDP classifier method.^6^

**Supplementary References**

1. Caporaso JG, Lauber CL, Walters WA, et al. Global patterns of 16S rRNA diversity at a depth of millions of sequences per sample. *Proc Natl Acad Sci U S A.* 2011;108 Suppl 1:4516-4522.

2. Biesbroek G, Sanders EA, Roeselers G, et al. Deep sequencing analyses of low density microbial communities: working at the boundary of accurate microbiota detection. *PLoS One.* 2012;7(3):e32942.

3. Callahan BJ, McMurdie PJ, Rosen MJ, Han AW, Johnson AJA, Holmes SP. DADA2: High-resolution sample inference from Illumina amplicon data. *Nat Meth.* 2016;13(7):581-583.

4. R Development Core Team (2008). R: A language and environment for statistical computing. R Foundation for Statistical Computing, Vienna, Austria. ISBN 3-900051-07-0, URL <http://www.R-project.org>.

5. Kraemer JG, Ramette A, Aebi S, Oppliger A, Hilty M. Influence of pig farming on the human's nasal microbiota: The key role of the airborne microbial communities. *Appl Environ Microbiol.* 2018.

6. Wang Q, Garrity GM, Tiedje JM, Cole JR. Naïve Bayesian Classifier for Rapid Assignment of rRNA Sequences into the New Bacterial Taxonomy. *Applied and Environmental Microbiology.* 2007;73(16):5261-5267.

**Supplementary Figure 1**

**Dissimilarity** **in-between initial and day 4 oropharyngeal samples**

Distance between the two samples, represented per group. There was no statistical significant difference between any of the groups. Mean dissimilarity and standard errors are indicated for each group.


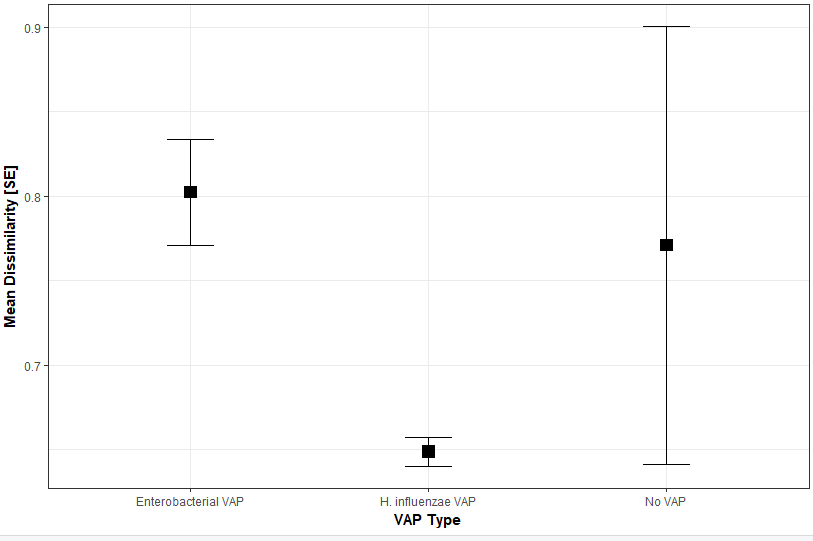


Abbreviations

VAP Ventilator-associated pneumonia

SE Standard error

**Supplementary Figure 2**

**Longitudinal alpha diversity in tracheobronchial samples**

Longitudinal course of diversity (Richness) of the oropharyngeal samples in the individual patients. Red=enterobacterial VAP, green= no VAP.

Statistics (linear-mixed effect model)

No VAP REF

Enterobacteriaceae -67.2 (SE +/- 13.4), p<0.001

H. influenzae -92.1 (SE +/- 13.4), p<0.001

Analysis of Variance Diag (d0) p=0.21

Abbreviations

VAP Ventilator-associated pneumonia


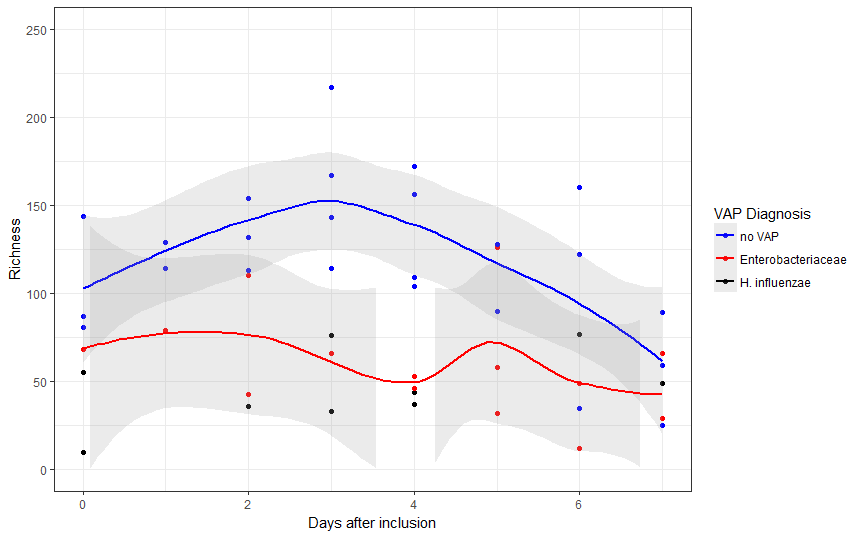

Supplement: Supplementary file 1 — a) Supplementary Methods (Microbiome); b) Supplementary References; c) Additional file 1: Figures S1 & S2. (DOCX 47 kb) [file 13756_2019_530_MOESM1_ESM.docx]
